# Avoidant restrictive food intake disorder: recent advances in neurobiology and treatment

**DOI:** 10.1186/s40337-024-01021-z

**Published:** 2024-06-07

**Authors:** Natasha K. O. Fonseca, Vitória D. Curtarelli, Juliana Bertoletti, Karla Azevedo, Tiago M. Cardinal, Júlia D. Moreira, Luciana C. Antunes

**Affiliations:** 1 Graduate Program in Psychiatry and Behavioral Sciences, Universidade Federal do Rio Grande do Sul (UFRGS), Ramiro Barcelos, 2400, Porto Alegre, RS 90035-003 Brazil; 2 Universidade Federal de Santa Catarina (UFSC), Florianópolis, SC Brazil; 3 Florianópolis, SC Brazil; 4 Laboratory of Neuroscience and Eating Behavior, Universidade Federal de Santa Catarina (UFSC), Florianópolis, SC Brazil; 5 Department of Nutrition, Health Sciences Center, Universidade Federal de Santa Catarina (UFSC), Florianópolis, SC Brazil; 6 Translational Nutritional Neuroscience Working Group, Universidade Federal de Santa Catarina (UFSC), Florianópolis, SC Brazil

**Keywords:** Avoidant restrictive food intake disorder, Eating disorders, Restrictive eating, Selective eating

## Abstract

Avoidant restrictive food intake disorder (ARFID) is an eating disorder characterized by persistent insufficient nutritional and/or energy intake. ARFID, before referred to as “selective eating disorder”, was introduced recently in the DSM-5 as a replacement for and expansion of the previous diagnosis. Individuals with ARFID may limit food variety and intake due to avoidance based on the sensory characteristics of the food or related to any adverse consequences of eating without the intention of losing weight and concerns of body image. The limited understanding of avoidant and restrictive eating poses challenges to effective treatment and management, impacting directly on the growth and development of children and adolescents. The ARFID neurobiological concept has not yet been clearly defined to clinical practice for nutritionists, thereby hindering screening and impeding the development of treatment recommendations. This narrative review provide useful practical information to consult the pathophysiology, the neurobiology, the clinical features, the assessment and the treatment for healthcare professionals seeking to enhance their clinical knowledge and management of this disorder.

## Abstract (plain language summary)

Avoidant restrictive food intake disorder (ARFID) is an eating disorder characterized by persistent insufficient nutritional and/or energy intake. Individuals with ARFID exhibit limited food intake and variety, often due to a lack in eating, without the primary goal of weight loss. The limited understanding of avoidant and restrictive eating poses challenges in terms of effective treatment and management, which directly impacts the growth and development of children and adolescents, as well as their nutrition and psychosocial well-being. ARFID is a relatively recent diagnostic classification, representing a burgeoning field of study. The identification of diagnostic criteria and the pursuit of new knowledge in this area have only recently begun. Consequently, assessment tools and treatment strategies are still in the process of development and validation. This narrative review explored the neurobiological perspective of ARFID using the three-dimensional model, examined its etiology and risk factors, evaluated clinical screening and evaluation tools, discussed common clinical complications, and presented different types of nutritional, behavioural, and pharmacological interventions used in ARFID treatment.

## Introduction

Eating behaviour relies on the successful integration of various neurobiological functions, as well as family and interpersonal relationships, during early human development. Disruptions in one or more of these multisystemic areas can lead to dysfunctional eating behaviours or eating disorders [1]. When a specific aspect of eating behaviour deviates from the norm or cultural norms and causes suffering, disability, and impairment in daily activities, it is highly likely to be classified as an eating disorder [2]. Eating disorders have a multifactorial etiology determined by genetic, sociocultural predispositions, biological and psychological vulnerabilities and patterns of interpersonal relationships [3].

Individuals with Avoidant Restrictive Food Intake Disorder (ARFID) may restrict their food intake and variety due to an apparent lack of interest in eating (low interest), exhibit avoidant eating behaviour based on sensory characteristics of foods (sensory limitations) and/or express concern about the aversive consequences of eating (fear) [4].

Studies on the neurobiological basis of ARFID support a three-dimensional model that explains the three primary clinical presentations: neurobiological alterations in sensory perception, appetite homeostasis, and negative valence systems. These alterations can create a negative and stressful environment surrounding food intake [5, 6].

## Methods

To synthesize recent advancements in understanding ARFID and identify current gaps in the literature, we conducted a comprehensive literature review using the MedLINE/PubMed and Scopus databases until April 2023. We selected all articles published in English and Portuguese since 2013. The keywords and terms employed in the review encompassed the following: Avoidant Restrictive Food Intake Disorder/ARFID; ARFID and Selective eating; ARFID and sensory sensitivity; ARFID and sensory hypersensitivity; ARFID Neurobiology; ARFID complication; ARFID treatment. Additionally, we actively searched for recently published meta-analyses and systematic reviews, as well as examined the reference lists of research articles for further relevant sources.

## Avoidant restrictive food intake disorder

Prior to the DSM-5, it became evident that a subset of children, adolescents, and young adults experienced eating difficulties that did not fit into existing diagnostic categories. These patients often received various diagnoses, including the residual diagnosis of Eating Disorder Unspecified [7, 8], which was inadequate as these individuals did not exhibit weight and/or shape concerns [1]. ARFID is a comprehensive term encompassing a range of eating-related issues, including apparent lack of interest in eating, selective and demanding eating, food phobia, avoidance based on sensory characteristics of food, emetophobia, functional dysphagia, and globus hystericus, which refers to anxiety about the aversive consequences of eating [8–10].

Initially, the diagnostic category was limited to children under the age of 6 and focused on the dysfunctional relationship between the eating disorder and caregiver-child interactions [1]. It is common for children to exhibit food-related complaints such as selectivity or avoidance during their developmental stages. They may have idiosyncratic preferences for specific flavours and consistencies, but these are typically self-limiting inclinations that are part of natural and transient development, seldom necessitating intervention [11, 12]. However, when a persistent condition arises, causing clinically significant problems and interfering with physical, social, and emotional development, the diagnosis of ARFID should be considered.

### Definition and diagnostic criteria

ARFID is characterised as a persistent eating disorder that exhibits clinical heterogeneity. According to the DSM-5, ARFID is defined by the following criteria: Criterion A—an eating disorder characterized by the persistent failure to meet appropriate nutritional and/or energy needs, resulting in (i) clinically significant weight loss, or, in the case of children, failure to achieve expected and adequate growth or delayed growth; (ii) significant nutritional deficiencies; (iii) reliance on enteral or oral nutritional supplementation; and/or (iv) substantial impairment in psychosocial functioning [13]. Criterion B states that the eating disorder cannot be better explained by the unavailability of food or a culturally accepted practice. Criterion C specifies that the eating disorder should not occur exclusively during Anorexia Nervosa (AN) or Bulimia Nervosa (BN), and there should be no evidence of disturbance in the perception of weight or body shape. Criterion D emphasizes that food restriction/avoidance should not be better explained by or co-occur with another medical condition or eating disorder. The eating disorder is not attributable to a co-occurring medical condition or better explained by another mental disorder, such as AN. When an eating disorder occurs in the context of another condition or disorder, its severity exceeds that usually associated with the condition or disorder and warrants additional clinical attention [13].

Restriction by the sensory characteristics of foods tends to appear in the first decade of child development and can last throughout adult life [13]. ARFID can have a chronic course that significantly impacts the psychosocial functioning of both the patient and their family. The diagnosis of ARFID is made when the individual fails to meet appropriate nutritional requirements (such as anemia and vitamin deficiency), resulting in significant weight loss that they are not influenced or motivated by body shape, lack of weight gain or growth in children, or when the behaviour markedly interferes with psychosocial functioning [10, 14].

### Clinical characteristics

Three subtypes were identified based on motivations for food avoidance and corresponding to the descriptive presentations of ARFID, according to the DSM-5: (1) ARFID-low appetite, individuals with limited food intake who had low appetite, lack of interest in food; had difficulties with the act of eating, such as small bites, prolonged time to finish meals; (2) ARFID-sensory limitations, those with limited variety associated with sensory problems and aversions related to certain foods or profound rigidity involving the act of eating such as food selectivity, food neophobia; and (3) ARFID-aversive, individuals with avoidance histories/nutritional restriction that occurred and/or evolved as a result of an event (choking, vomiting) or specific fear (fear of choking, pain or nausea).

However, although the DSM-5 criteria include these commonly observed clinical presentations of ARFID, there is a lack of evidence supporting the etiology behind these presentations. Thus, these clinical subtypes serve as illustrative examples to aid in accurate diagnosis, recognizing that other causal processes may contribute to food restriction in children with ARFID [15].

A strategy used in previous studies to understand the clinical presentation of patients with ARFID has been to compare them with cases of AN and BN [7, 16, 17]. When compared with individuals with AN or BN, individuals diagnosed with ARFID tended to be significantly younger (mean age in ARFID ranged from 11.1 to 14.6 years versus 14–15.6 years in AN and 14.9– 16.7 years in BN), have a longer duration of illness (12–33 months in ARFID vs. 8–23 months in other EDs) and have more male patients (men ranging from 21 to 50%); although female patients continue to be the majority among all diagnostic groups and, in general, the pediatric sample with ARFID is equally common in boys and girls [18]. Patients with ARFID were more likely to have a medical condition or an anxiety disorder compared to patients with AN or BN, however, they were less likely to have mood disorders [7, 16]. In terms of weight, patients with ARFID consistently are at a lower weight than patients with BN, but at a similar weight or a slightly higher weight compared to patients with AN [7, 15, 17, 19]. Patients with ARFID, during admission interviews for daily programs targeting eating disorders, report no body image distortion or desire for weight loss. They also exhibit fewer compensatory behaviors commonly associated with eating disorders, such as purging or excessive exercise [16].

### Etiology and risk factors

It is important to highlight that the majority of the available knowledge on ARFID is based on studies involving relatively small clinical samples, primarily comprising individuals from eating disorder programs or those seeking specialized medical help [15]. Furthermore, due to the relatively recent establishment of diagnostic criteria, investigations into the prevalence and etiology of ARFID have mainly relied on retrospective studies [7, 16, 17, 19, 20]. Another knowledge gap lies in understanding the characteristics associated with ARFID in adults, as most studies have focused on pediatric samples covering childhood and adolescence [21]. Even without exact data, a prevalence range of 0.5–5% is estimated in the general population, both in adults and children [22]. The incidence of ARFID in children 5 to 18 years of age was 2.02 (95% CI 1.76–2.31) per 100.000 patients in a study with 207 children and adolescents based on the Canadian Paediatric Surveillance Program survey [23]. In this same study, Kaztman et al. (2021) show that age- and sex-specific differences were noted for diagnostic criteria, medical characteristics, psychiatric comorbidities, eating behaviors, and hospitalization [23].

ARFID has a heterogeneous group of presentations with likely multiple etiologies, such as neurobiological, neurodevelopment, genetic, sociobehavioral among others. Most individuals diagnosed with ARFID have primary medical diagnoses comorbid food difficulty and restriction, most commonly neurological or gastroenterological disorders. Nonetheless, even when associated, these disorders are not considered the reason for the development of ARFID, as Criterion D states that the eating behavior is not better explained by something else.

Neurodevelopmental disorders are comorbid some presentations and are more common especially Autism Spectrum Disorder (ASD) and Attention Deficit Hyperactivity Disorder (ADHD). [14]. Patients with comorbid autism spectrum disorder (ASD; 28%) showed more food-related sensory sensitivities (RR = 1.26) and greater lack of interest in eating (RR = 1.18) than those of patients without ASD (49%) [24]. While other patients may or may not have another associated medical condition. Patients' anxiety traits showed the greatest positive correlations with symptoms of concern about aversive consequences of eating [24]. Certain presentations of ARFID bear similarities to anxiety disorders (e.g., choking phobia) and are likely to have a similar etiology (psychiatric and general medical factors are involved and may be a response to environmental stressors) [14].

Zickgraf et al. (2019) suggests that etiological and maintenance factors that influence eating behavior in ARFID may be associated with distinct patterns of restrictive eating [25]. While Thomas et al. (2017) hypothesize that the clinical presentation of an individual with ARFID can be understood as a single point along a three-dimensional space, that is, the three presentations described above vary in severity but are not mutually exclusive (sensory perception, homeostatic appetite and negative valence systems). The available data supporting the dimensional model suggest that the majority of diagnosed individuals undergoing psychological treatment present eating difficulties in several domains of the ARFID [6].

**Fig. 1:**
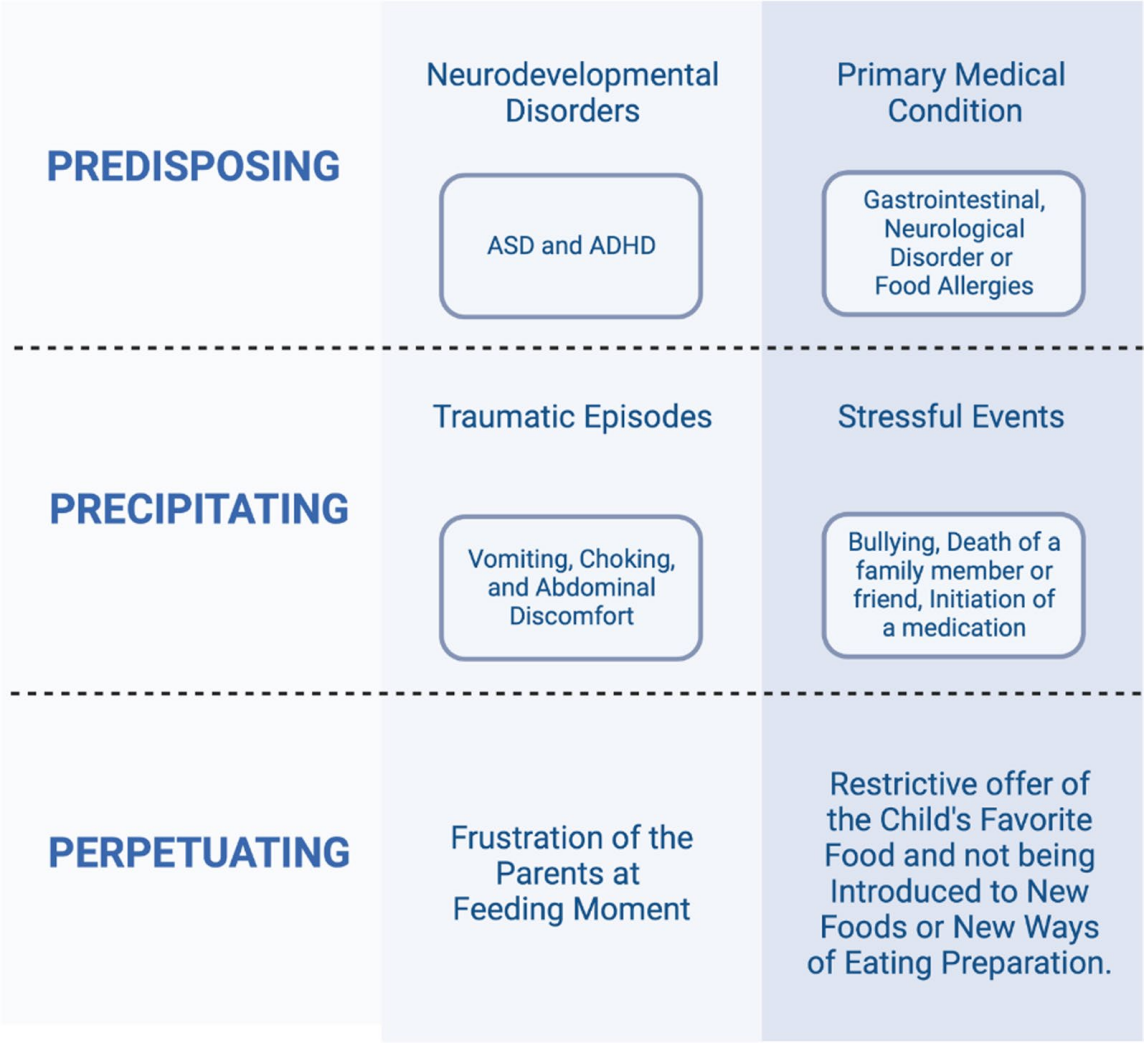
Predisposing, precipitating, and perpetuating factors of the ARFID framework

According to Brigham et al. (2018), the etiology of ARFID may involve biological factors, sensory sensitivity, anxiety traits, and the interplay between homeostatic and hedonic aspects of eating behaviour, which can contribute to increased vulnerability and act as predisposing factors [26]. Coglan and Otasowie (2019) propose a framework for understanding the contributing factors to ARFID, categorizing them into three groups, similar to models proposed for other eating disorders: predisposing, precipitating, and perpetuating factors [10] (Fig. 1).

Predisposing factors encompass neurodevelopmental disorders, such as ASD and ADHD, which serve as the foundation for certain clinical presentations of ARFID. Sensory hypersensitivity, often observed in individuals with ASD, can interfere with the consumption of specific foods, resulting in dietary restrictions. The distractibility and agitation associated with ADHD can impact eating behaviour, leading to decreased interest in food and heightened arousal levels during mealtimes. Additionally, a primary medical condition can act as a predisposing factor, as a significant proportion of children with feeding difficulties have gastrointestinal or neurological disorders, as well as food allergies [10].

Among the precipitating factors are traumatic episodes or some specific event in which individual has been experienced or witnessed such as vomiting, choking, and abdominal discomfort. Stressful events that may contributed on the pathogenesis of eating disorders depends on how each individual reacts to situations and the resources that each one has to respond to them [4].

A previous study retrospectively determined the incidence of ARFID in children and adolescents using the DSM-5 diagnostic criteria in a pediatric eating disorder program [27]. The authors observed that 71.4% of patients reported a triggering factor for their eating disorder, including abdominal pain, bullying, death of a family member or friend, initiation of a medication, vomiting or witnessing vomiting, food allergy concern, and animal rights concern [27].

Among the factors that perpetuate or maintain the ARFID are the frustration of the parents at feeding moment; the restrictive offer of the child's favorite foods; and not being introduced to new foods or new ways of eating preparation. All these examples are recognized for contributing to children's feeding problems and may be one of the explanations for the maintenance of dysfunctional behaviors associated with the disorder [10, 16].

## Neurobiology of ARFID

**Fig. 2:**
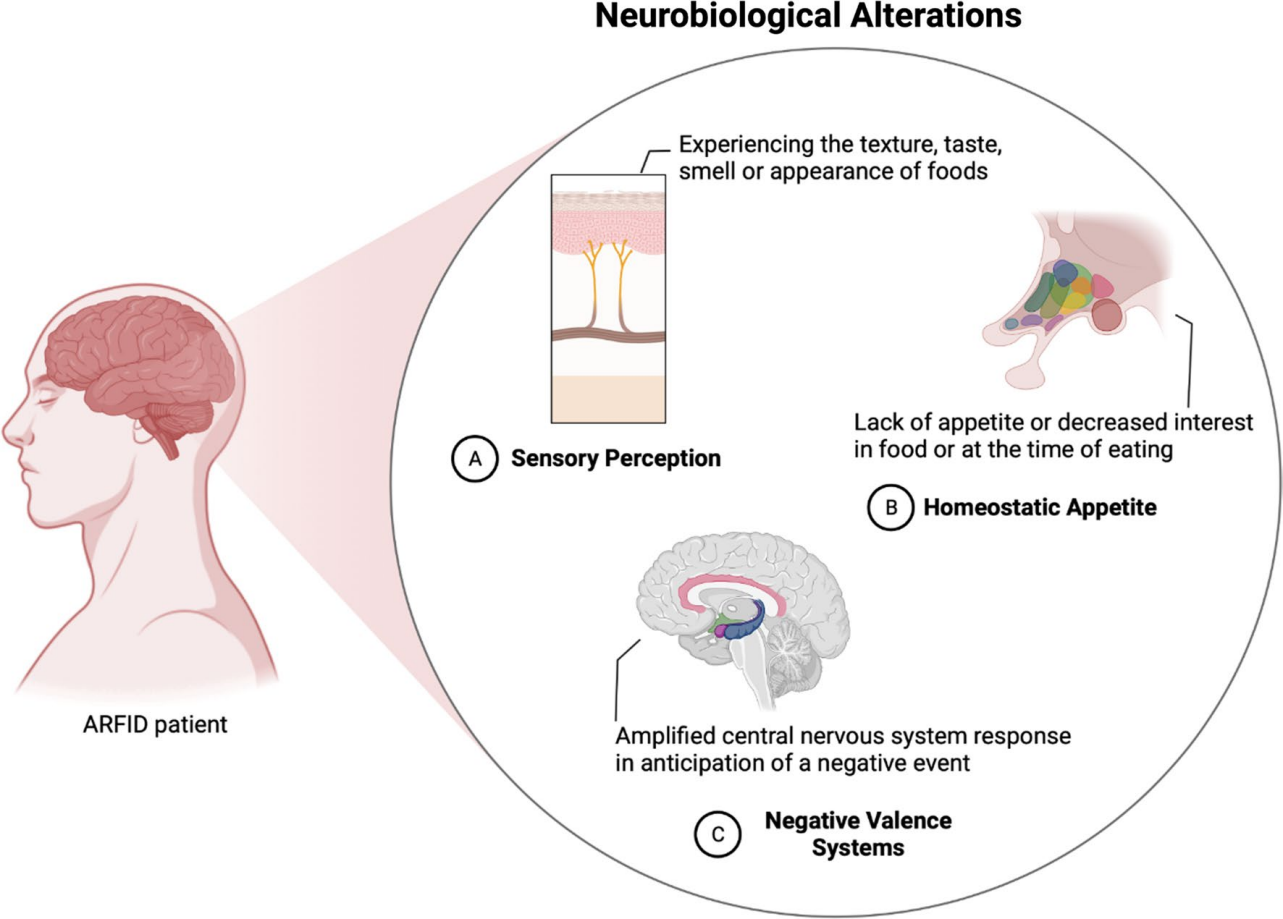
The neurobiological basis of ARFID

Various studies have examined the neurobiological basis of ARFID (Fig. 2). Patients with ARFID often exhibit restrictive eating behaviours that can be categorized into different functional domains described below [5].

### ARFID and sensory sensitivity

Individuals with ARFID who exhibit food avoidance behaviour based on discomfort associated with the sensory properties of food (such as texture, temperature, colour, smell, and presentation) often report intensely negative taste experiences with non-preferred foods [6, 13, 28, 29]. This domain of ARFID involves the selective refusal of specific food items while accepting preferred foods without difficulty [30].

Sensory sensitivity, considered an intrinsic characteristic, involves differences in the perception, reactivity, and integration of sensory information, including taste (bitterness, sweetness), touch sensations (viscosity, food texture), and odours, which vary among individuals. Sensory sensitivity and sensory integration disorders have been associated with food selectivity [7, 31].

Hyperresponsiveness or hypersensitivity refers to an exaggerated response to sensory stimuli. In individuals with ASD, food selectivity may be related to sensory sensitivity, sensory processing dysfunction, and altered responses to sensory inputs, particularly in terms of sensory modulation, resulting in hyper or hyporesponsiveness [32, 33]. Both food avoidance and food selectivity are commonly observed in individuals diagnosed with ASD and ADHD. Parents and clinicians frequently report that children with ASD exhibit highly selective eating patterns and have a limited variety of foods in their diet [32, 34]. In these cases, sensory hyperresponsiveness appears to be associated with food selectivity [33].

On average, 13–50% of patients with ARFID present comorbidities with ASD [35]. Bourne and collaborators (2022) state that although the literature on children and young people with autism reliably highlights the three main reasons for food avoidance and restriction in ARFID, according to the original diagnostic guidelines, sensory sensitivities are currently the most cited. Given the atypical sensory processing associated with ASD, texture aversion is the most commonly reported concern, although sensitivity to taste, temperature, type, color, and appearance have also been described [35].

One consistent feature of ASD is that it is more commonly diagnosed in boys than in girls [36]. On the other side, Katzman et al. (2021) show that boys had a higher rate of refusal based on sensory characteristics (51.2%; 95% CI 40.2–62.2%) compared with girls (31.5%; 95% CI 23.9–40.2%; *P* = 0.007) in a sample of 207 children and adolescents with ARFID [23]. The DSM-V shares that ARFID is more common in men than women in those with comorbid ASD. What still remains a gap in the literature is whether the sensory profile would be greater in men than in women and whether sex could predict this characteristic of sensory sensitivity.

Thomas et al. (2017) hypothesized that sensory hypersensitivity influences taste perception [6]. A study by Kauer et al. (2015) found that adults self-identified as "picky eaters" were more likely to reject foods with bitter or sour tastes but not those that were sweet or salty [37]. Moreover, they rated sweet and bitter flavours as more intense compared to the control group. Individuals who were more sensitive to bitterness were referred to as "supertasters" and exhibited aversive reactions and subsequent refusal of foods [30]. Research on food selectivity in children suggests that food avoidance may be based on hypersensitivity to food texture, indicating that texture can influence flavour perception. Children with sensory defensiveness, particularly in the tactile domain, exhibit aversion and high sensitivity to specific textures and consistencies of food [37, 38]. The rate of refusal based on sensory characteristics (5–9 years of age: 66.7%; 95% CI 47.9–81.3%; 10–14 years of age: 38.6%; 95% CI 30.7–47.3%; 15–18 years of age: 22.2%; 95% CI 12.3–36.9%; *P* < 0.001) was higher among younger children whit ARFID [23].

Individuals with sensory hyperresponsiveness exhibit faster, more intense, and longer-lasting responses to sensory stimuli compared to those with typical sensory responsiveness. This non-gradual, maladaptive response is postulated to stem from difficulties in regulating and organizing the degree and intensity of sensory input response in individuals with sensory modulation dysfunction [32, 33, 39]. Christol et al. (2018) explored the hypothesis that patients with oral sensory hypersensitivity exhibit structural differences in brain areas associated with taste perception [32]. Children with sensory food aversions (SFAs), commonly referred to as "picky eaters" or "selective eaters," also display hypersensitivity to sensory experiences beyond meal contexts [30].

Genetic studies have revealed an inherited pattern of food sensitivity, particularly concerning sensitivity to the bitter taste of 6-n-propylthiouracil (PROP), which influences food choices. Certain polymorphisms in the taste receptor gene family, specifically the Tas2r gene, have been found to differentiate "supertasters" from "non-tasters" [30, 40, 41]. Selective eating associated with hypersensitivity in taste perception may co-occur with the ARFID domain of sensory avoidance and fear of aversive consequences, where an extreme aversive reaction to a particular food can result in nausea or vomiting [30].

### ARFID and inappetence

Food restriction or avoidance is characterized by a general lack of interest in food and eating, low appetite, early satiety, difficulties with the physical act of eating (such as taking small bites or pieces), prolonged mealtime, reporting a lack of hunger at mealtimes, forgetting to eat, or feeling full faster than others [13, 28]. According to the hypothesis proposed by Thomas et al. (2017), this clinical presentation of ARFID may be associated with differences in the activation of regulatory centers involved in hunger and satiety perception [6].

The initiation of a meal is influenced by various factors, including external or non-homeostatic factors such as social situations or time constraints, as well as internal or homeostatic factors related to energy substrates, hunger, and satiety [42]. Homeostatic regulation involves the integration of hormonal and metabolic signals with information from the gastrointestinal tract and autonomic nervous system activity in the brainstem and hypothalamus, resulting in a coordinated regulatory response for energy homeostasis [43, 44]. The hypothalamus and insular cortex play crucial roles in integrating hunger and satiety signals. Hunger appears to increase hypothalamic activity, while satiety exerts an inhibitory effect [42, 45]. Distinct centers responsible for appetite and satiety have been identified: the lateral hypothalamus (LH) regulates the termination of feeding, whereas the paraventricular nucleus of the hypothalamus (PVN) is associated with the initiation of feeding [46, 47].

Thomas et al. (2017) discussed the neurobiology of individuals exhibiting inappetence or low interest in food, proposing that there may be a distinct pattern of activation in the centers responsible for regulating hunger and satiety perception [6]. The findings of a study by Kerem et al. (2022) support the existence of divergent neurobiological foundations for restrictive/avoidant eating behaviour in individuals with ARFID and body mass index (BMI) associations [48]. The authors identified significant hyperactivation in the brain regions of the orbitofrontal cortex and anterior insula (regions associated with food anticipation and reward processing in response to visual cues of palatable food) in fasted individuals with ARFID and overweight/obesity, compared to individuals with ARFID and normal weight. However, it is important to question the directional relationship of these findings, namely whether such changes result from the neurobiology of a specific clinical subtype of ARFID or if they are consequences of the nutritional status impairment in these patients.

Nevertheless, there is no consensus regarding the plausibility of homeostatic dysregulation being associated with the acute onset of restrictive behaviour explained by inappetence, while deficits in reward sensitivity have been found in other eating disorders, particularly AN [25].

According to neuroendocrine findings, the literature shows that this patients with ARFID may to have distinct patterns of secretion of gut-derived appetite-regulating hormones, especially those have a low-weight, compare to AN and healthy controls. Higher levels of fasting ghrelin, high anorexigenic peptide YY (PYY) levels post-meal, higher levels of plasma fasting cholecystokinin (CCK) and higher basal or post-prandial levels of glucagon-like peptide 1 (GLP-1) are hypotheses explored in these patients, such as in AN [49]. Becker et al. (2021) show low-weight ARFID showed lower levels of total ghrelin around a meal than AN, low-weight ARFID did not differ from AN or healthy controls in PYY levels and did not show sustained high PYY levels post-meal [50]. Murray et al. (2022) found fasting CCK was higher in those with full/subthreshold ARFID versus healthy controls with a large effect, controlling for age, sex, and BMI percentile [51]. Aulinas et al. (2020) also observed distinct medical and endocrine alterations in ARFID compared to AN, such as a lower number of menses missed, higher total T3 levels, and lower total T4: total T3 ratio [52]. Neuroendocrine changes must be highly interconnected with homeostatic dysregulation and must be further studied in the future, especially in research with weight recovery, to assess whether there is normalization in neuroendocrine levels, and thus identify whether this change is part of the pathophysiology of the disorder or directly associated with malnutrition. Better understanding this mechanism can assist in differential diagnosis, in addition to providing new treatment targets.

### ARFID and fear of aversive consequences of eating

Restrictive avoidant eating behaviour may arise as a conditioned negative response linked to the fear of experiencing aversive consequences while eating, following a traumatic event or specific fear. The ARFID subdomain known as the "fear of aversive consequences" typically manifests suddenly after a traumatic incident that resulted in unpleasant outcomes such as pain, nausea, choking, vomiting, or invasive clinical procedures involving the gastrointestinal tract (e.g., upper digestive endoscopy, esophagoscopy) [13].

Individuals who have undergone food-related trauma and subsequently develop avoidance behaviours to protect themselves from negative experiences may exhibit restrictive avoidant eating behaviour [26, 28, 30]. Any aversive encounter associated with food can trigger a conditioned fear of eating in susceptible individuals. Several patterns can be observed that contribute to this restriction, including the fear of eating after a single traumatic event, such as a choking incident; fear of eating in children who have undergone painful or unpleasant oral procedures; and fear in children who were tube-fed, lacked significant feeding milestones, or had limited experiences with food, leading to a sense of threat when food is presented orally [53].

According to Brigham et al. (2018), individuals within this domain of ARFID often possess an inherent predisposition to anxiety, resulting in the generalization of food avoidance beyond the initial target food. This may extend to similar foods, entire food groups, or environments that evoke memories of the traumatic event [26]. Thomas et al. (2017) propose hypotheses regarding the neurobiological mechanisms associated with the ARFID subdomain of fear of aversive consequences, suggesting a possible hyperactivation of brain circuits involving the amygdala and anterior cingulate cortex, which are key structures in the limbic system involved in fear processing, or hyperactivation of the defence system [6, 21].

Anxiety plays a prominent role in theoretical models of avoidant eating behaviour, with previous studies indicating elevated anxiety symptoms in children with ARFID [54]. Conceptually, fear and anxiety can be understood as brain states triggered by external or internal stimuli, giving rise to specific measurable behavioural, physiological, hormonal, and autonomic reactions (55). Fear and anxiety elicit defensive behavioural responses that evolved to enable organisms to avoid or mitigate harm and ensure survival—a vital adaptation mechanism in potentially hostile environments. However, in humans, excessive fear and chronic anxiety transition from adaptive responses to maladaptive ones, causing damage and dysfunction [55].

Previous studies have emphasized the involvement of specific brain regions in generating fear and anxiety, as well as the contribution of synaptic and neuromodulatory processes within these regions. Indeed, studies using the Pavlovian model of conditioned fear have revealed the existence of a network distributed across brain regions involved in fear learning and expression. These structures include but are not limited to, the amygdala, medial prefrontal cortex (mPFC), and hippocampus [55]. One fundamental principle of fear learning is the requirement for activity-dependent plasticity within the amygdala. Sensory inputs from various modalities converge in the lateral amygdala (LA), receiving auditory, visual, and somatosensory information related to conditioned and unconditioned stimuli. Plasticity in the LA, induced by conditioning, precedes that in the cortex and thalamus, develops more rapidly than the conditioned behavioural response, and is believed to drive the expression of conditioned fear behaviour [55].

Specific phobias entail intense and persistent fears of certain objects or specific situations, which often result in avoidant behaviour. On the other hand, Post-Traumatic Stress Disorder (PTSD) arises from a traumatic event. However, it is important to note that specific phobias can either stem from a traumatic event (experimental phobia) or have no experiential basis (non-experiential phobia) [56–58]. The individual's response to trauma depends not only on the characteristics of the event or stressor but also on specific factors unique to the individual, such as genetic or experiential predispositions [59]. In light of this, Thomas et al. (2017) suggests that a subgroup of individuals who develop ARFID after a traumatic eating experience may have had a pre-existing vulnerability, which contributes to an increased phobic response [6].

Chatoor, Conley, and Dickson (1988) conducted a review of cases involving children who exhibited food refusal and acute onset of food-related anxiety after episodes of choking. These children also experienced intense anticipatory eating anxiety. The study termed this phenomenon "Post Traumatic Eating Disorder" since the children's reactions to the aversive episode resembled those of individuals who had experienced a traumatic event and developed PTSD [30].

Behaviours associated with specific experiential phobias can be sustained and perpetuated by dysfunctions in the fear mechanism that is dependent on learning. This can include impairments in fear extinction and a lack of habituation to fear, which refers to the failure to acquire a reduced fear response through repeated exposure to the fear-inducing stimulus. The maintenance of specific experiential phobias can be attributed to operant conditioning of fear, which reinforces avoidance behaviours [56]. In the case of ARFID, children may avoid the target food or mealtime to escape the discomfort or anxiety it generates, thus negatively reinforcing the avoidance behaviours and perpetuating the eating psychopathology [60]. However, unlike the effectiveness observed in exposure-based treatments for patients with anxiety disorders [61], daily exposure to the visual, olfactory, and harmless consequences of consuming food alone does not appear to reduce avoidant behaviour in individuals with ARFID [62, 63]. Potential hypotheses as to why exposures do not improve food selectivity in ARFID may include cognitive factors that impede experiential learning [64].

Another crucial factor to consider is the role of repulsion in the manifestation of anxious symptoms in individuals with ARFID. The relationship between repulsion and anxiety offers new insights, as disgust is functionally associated with preventing contamination by pathogens (as may occur with the ingestion of contaminated food) and is resistant to extinction mechanisms. An exploratory study involving 1,644 adults analysed the contributions of sensory sensitivity, anxiety, and the experience of disgust to elucidate the potential role of disgust in food avoidance within ARFID. The findings revealed that disgust fully mediated the association between anxiety and ARFID. Thus, repulsion may play a significant role in food avoidance and could provide an explanation for novel approaches towards more effective treatments.

## Clinical screening and evaluation tools of ARFID

The identification of ARFID as a diagnostic entity is relatively new, and currently, there is a lack of a standard assessment tool for its specific psychopathology. This gap hampers the identification of ARFID in clinical settings, understanding its etiology and associated risk factors, assessing the effectiveness of treatment, and verifying the epidemiology and natural course of the disorder [12]. It has been observed that many psychometric measures commonly used in eating disorder contexts are not specific enough to facilitate a diagnosis of ARFID, thereby resulting in low sensitivity if used as assessment tools [27]. However, screening tools for ARFID are in the early stages of development and validation.

Several screening and diagnostic instruments have been developed, which can be divided into assessments structured clinical interviews (EDA-5, Eating Disorder Assessment for DSM-5; SCID-5, Structured Clinical Interview for DSM-5; PARDI, Pica, ARFID, and Rumination Disorder Interview; EDE, Eating Disorder Examination) and self-reported questionnaires (EDY-Q, Eating Disorders in Youth-Questionnaire; NIAS, Nine-Item ARFID Screen; FNS, Food Neophobia Scale; PARDI-AR-Q, Pica, ARFID, and Rumination Disorder – ARFID Questionnaire) to evaluate specific type of use for clinicians and researchers [15, 65].

### *Pica*, ARFID and rumination disorder interview (PARDI)

Bryant-Waugh et al. (2019) evaluated the feasibility, acceptability, reliability, and validity of the PARDI's psychometric properties [66]. PARDI is a structured interview developed to assess the presence and severity of diagnoses for evaluation and treatment planning in clinical and research settings, specifically focusing on ARFID. It encompasses the clinical presentations of the three phenotypes of ARFID, namely sensory sensitivity, lack of interest in eating, and fear of aversive consequences. These distinct explanations for dietary restriction may require different treatment approaches. PARDI shows promise as a tool, with initial data indicating good feasibility, adequate acceptability, and good internal consistency for the three-dimensional phenotypes of ARFID. Internal consistency of the three ARFID profiles was in the adequate to good range, with Cronbach’s alphas as follows: sensory sensitivity (0.77), lack of interest food or eating (0.89), fear of aversive consequences (0.79), and overall severity (0.89). Cohen’s κ for the ARFID diagnosis (coded as yes or no) was 0.75. However, clinical measures for validation and convergence between self-report and parent/caregiver reporting have not been examined [66].

### Eating disorder examination (EDE-ARFID)

In a similar study, Schmidt et al. (2019) presented a pilot study proposing the inclusion of ARFID criteria in the children's and parents' version of the Eating Disorder Examination (EDE), referred to as EDE-ARFID [67]. Based on 22 items rated on a 7-point Likert scale (0 = never/not at all to 6 = every day/extremely), the EDE subscales restraint, eating concern, weight concern, and shape concern are built, with higher subscale and global mean scores indicating more eating disorder psychopathology over the past 28 days. For this study, the subscales weight and shape concern and the global score were used, with Cronbach's α in this study of 0.81, 0.94, and 0.94 for the EDE [68]. In a non-clinical sample of children aged 8 to 13 years with low weight and/or restrictive eating behaviors, the EDE-ARFID module demonstrated high reliability, convergent and divergent validity, and the ability to distinguish between children with and without ARFID based on anthropometric and clinical characteristics [67]. The EDE-ARFID tool serves as both a diagnostic instrument and a means to collect clinical information related to the psychopathology of ARFID [15, 67, 69].

### Eating disorders in youth-questionnaire (EDY-Q)

The EDY-Q [70] is a self-report tool consisting of 14 items designed to detect early-onset eating disorders in children aged 8 to 13 years. The total mean score had an internal consistency of Cronbach's α = 0.62, reflecting heterogeneity of EDY-Q items [70]. Twelve of these items specifically address the symptoms of ARFID, including the three variants proposed by the DSM-5 [13]. The EDY-Q focuses on assessing restrictive eating behaviors while excluding concerns about weight or shape. It can be utilized as a quick screening tool for ARFID-related symptoms [15, 70]. Other tools such as the Behavioral Pediatrics Feeding Assessment Scale (BPFAS) and the Child Food Neophobia Scale (CFNS) can also aid in distinguishing ARFID from demanding eating during normal development in younger children [71].

### Nine item ARFID screen (NIAS)

Zickgraf and Ellis (2019) conducted a validation study of the NIAS, a brief, multidimensional, self-report screening tool developed to assess selective and restrictive eating behaviors in adults. The NIAS addresses food restriction behaviors associated with the three eating patterns related to ARFID, namely appetite, fear, and sensory sensitivity. The results from a non-clinical sample of adult college students and a sample of adults potentially at risk for ARFID support the use of the NIAS to assess restrictive eating behavior, characterized by picky eating, lack of appetite or limited interest in eating, and fear of negative consequences from eating [25]. Acceptable Cronbach's alpha and omega coefficients for the total scaleand subscales were obtained, in the entire sample. For the total scale α = 0.84, ω = 0.90; the selective/neophobic eating subscale was α = 0.77, ω = 0.77, while the appetite subscale was α = 0.74,ω = 0.70, and lastly, for fear subscale were α = 0.80, ω = 0.80 [72].

### Food neophobia scale (FNS)

The FNS is a 10-item measure that measures reluctance to try new foods [73]. This scale was originally validated for se in adults and has been adapted in children [74]. Higher scores on the FNS are indicative of behavioral responses to novel foods (e.g., eating fewer unfamiliar foods presented). Cronbach's alpha for the Food Neophobia Scale was 0.82, indicating a high degree of internal validity. Food neophobia scores were normally distributed with a mean ± SE of 29.6 ± 0.70 [75].

## Clinical complications in ARFID

The diagnosis of ARFID encompasses patients with diverse presentations, resulting in different clinical outcomes [13, 26, 76]. ARFID poses a significant impact on the variety, quality, and quantity of the diet, as it is associated with an inadequate composition of macronutrients and micronutrients. Consequently, there is an increased risk of nutritional deficiencies, improper linear growth, dependence on enteral nutrition and nutritional supplements, and the need for hospitalization to restore nutritional status [69, 77].

Regarding studies on individuals with food selectivity, the results do not consistently indicate a correlation between selectivity and low body weight [62, 78, 79]. However, some studies have reported weight loss [27, 80, 81]. There is no demonstrated association between food selectivity and overweight/obesity [79]. Individuals with ARFID commonly have a diet characterized by processed and refined carbohydrates, and foods with added sugars, with lower consumption of proteins, vegetables, and fruits. Compared to children without eating disorders, children diagnosed with ARFID exhibit lower levels of vitamin K (due to reduced vegetable consumption) and vitamin B12 (resulting from decreased consumption of animal proteins) [77, 82].

Similar to other eating disorders, ARFID can have profound clinical, nutritional, and psychosocial implications, particularly during childhood and adolescence. These implications may include severe limitations in food acceptance and reduced daily energy intake. Prepubertal infants, children, and adolescents diagnosed with ARFID may experience delays in typical development or growth, which can negatively impact their learning abilities [13, 60].

Patients with ARFID and growth deficits may require enteral nutrition or oral nutritional supplements to ensure adequate caloric and nutritional intake [69, 81], depending on the severity of the condition. ARFID shares some clinical complications, such as malnutrition, weight loss, or failure to gain weight due to restriction/avoidance, with AN [25, 71, 77]. However, compared to the clinical course of AN, patients diagnosed with ARFID tend to develop the eating disorder at an earlier age and require longer hospital stays for medical and nutritional stabilization [76, 83, 84]. Although patients with ARFID may have a similar BMI to those with AN, they typically experience less significant weight loss before treatment [76]. Furthermore, during hospitalization, patients with ARFID more frequently require enteral nutrition to meet their energy needs and exhibit less resistance and distress during the therapeutic process compared to those with AN [80, 81, 84].

In severe cases of ARFID, patients may display signs and symptoms of malnutrition, such as fatigue, lethargy, difficulty concentrating, memory deficits, presyncope, constipation, hypersensitivity to cold, hypothermia, electrolyte abnormalities (e.g., hypokalemia), dry skin, lanugo, alopecia, bradycardia, orthostatic postural tachycardia, hypotension, and a sunken abdomen. These symptoms are attributed to deficiencies in macronutrients and micronutrients [26, 69, 71, 85].

Moreover, female patients with ARFID may experience pubertal delay before the onset of menstruation. Primary amenorrhea may occur, and in postmenarcheal patients, secondary amenorrhea may result from low body weight. These findings are associated with lower bone mineral density [26, 69, 71, 85]. In cases of extreme food restriction, individuals may become malnourished and require medical stabilization in specialized inpatient units [40, 85].

Children with ARFID may exhibit impairments in recognizing satiety signals and have difficulty following appropriate eating habits. Those who reject foods that require extensive chewing, such as meats and tough vegetables, and only accept softer textures or pureed consistencies, may experience delays in oral motor development due to lack of experience with chewing. Additionally, they may also experience speech delays [1, 30].

In addition to significant clinical and nutritional complications, ARFID's selective and restricted eating patterns have a negative impact on social and emotional development, leading to higher levels of stress and family conflict in food-related contexts [13], as well as greater likelihood of comorbid neurodevelopmental disorders, disruptive behavior disorders and conduct disorders [86]. The severity of ARFID, particularly in terms of fear of aversive consequences related to eating, is associated with higher levels of anxiety, obsessive–compulsive disorder, and trauma-related disorders [86–88].

Undoubtedly, the presence of comorbidities has a more reserved course and prognosis. Children's initial knowledge about food is incomplete, and it is through experiences with caregivers, family members, and social interactions involving food that their knowledge is enhanced [7, 31]. Selectivity and extreme food avoidance can hinder a child's experiences in social settings, including interactions related to eating at school or with peers. The implications of this significant gap in child development may persist throughout the individual's lifespan.

## Treatment of ARFID

**Fig. 3:**
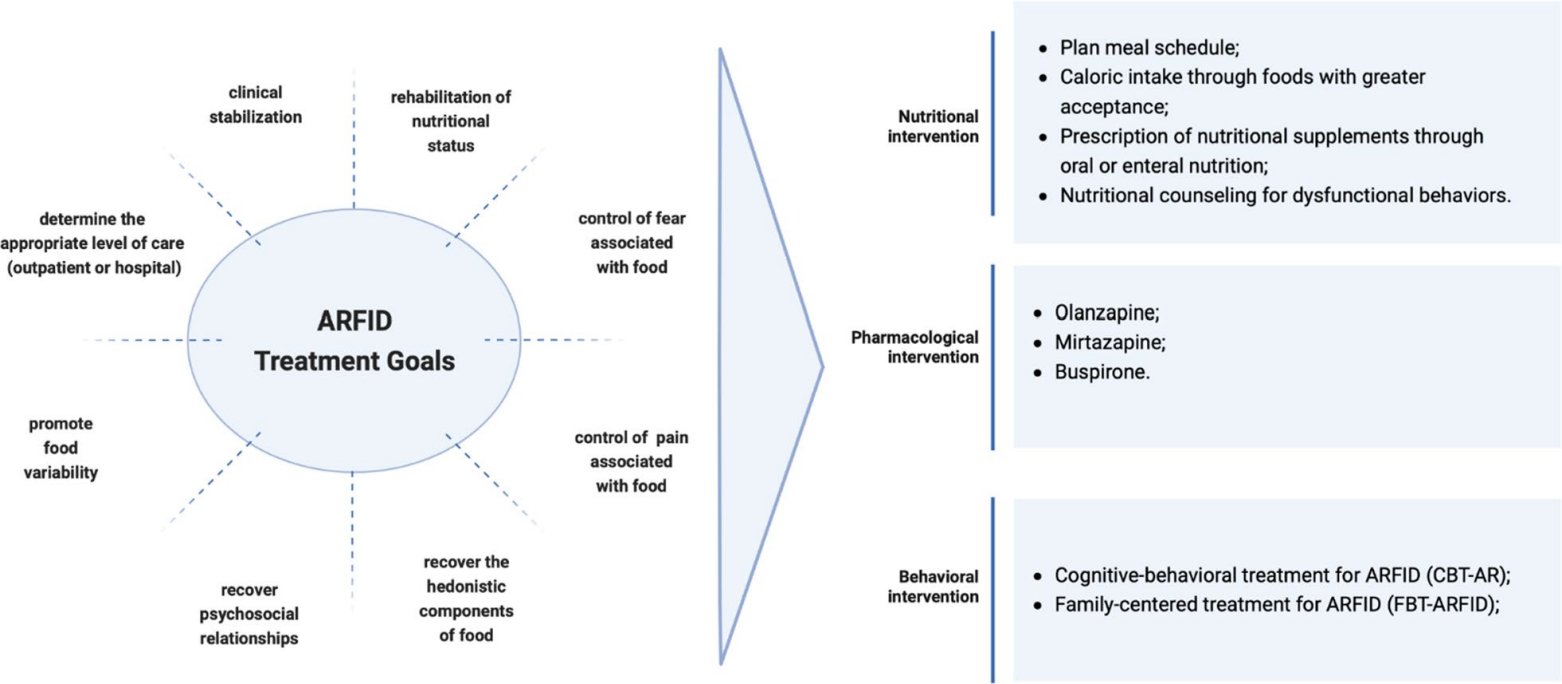
The treatment of ARFID

The management of ARFID (see Fig. 3) aims to determine the appropriate level of care, whether it be outpatient or in a hospital setting. The goals of treatment include clinical stabilization of the patient, restoration of nutritional status, addressing fear and/or pain associated with food, gradually expanding food variety, and facilitating the recovery of hedonistic aspects of food and psychosocial relationships related to eating [85]. Patients diagnosed with ARFID may not present just one of the domains. As such, it is important not to isolate patients into sub-profiles and treat them solely as “sensory” patients, for example, but rather to target personalized treatment to each individual.

There is limited evidence available to guide healthcare professionals in the treatment of ARFID, and no specific guidelines or consensus have been established. This lack of guidance can potentially lead to prolonged hospitalizations and the need for intensive care for complex cases [8, 26]. Like other eating disorders, the treatment of ARFID requires a multidisciplinary approach involving a team of healthcare professionals, ideally including paediatricians, psychiatrists, psychologists, nurses, nutritionists, and occupational therapists. This multidisciplinary team addresses all aspects of the individual's functioning [9, 89]. In many cases, patients with ARFID follow eating disorder programs that employ a range of strategies and approaches, similar to those used for other eating disorders, particularly AN. Treatment modalities can vary from outpatient care with a multidisciplinary team to hospitalization for close monitoring and rehabilitation of clinical and nutritional status [26, 89].

Therefore, given the heterogeneous clinical presentation of ARFID, the treatment plan for each patient should incorporate strategies tailored to their specific needs. It is essential to recognize that the requirements of patients with ARFID may differ based on the clinical presentation of the disorder and the presence of associated neuropsychiatric comorbidities. This approach allows for the identification of the precipitating and maintaining factors of restrictive eating behaviours and food avoidance. Moreover, understanding the dietary characteristics and associated clinical and psychiatric comorbidities is crucial, considering the complexity that can accompany the diagnosis [8, 90–92].

### Nutritional intervention in ARFID

The clinical manifestations of ARFID may result in impaired nutritional status, as indicated by insufficient weight gain, low body weight, growth deficits, and malnutrition. Addressing these complications is of utmost therapeutic importance and should be initiated promptly.

The primary objective is to restore body weight initially. Nutritional strategies employed for weight recovery and prevention of further weight loss should ensure adequate nutrient intake, particularly concerning calories. This can be achieved through the consumption of foods that are more readily accepted by the individual, as well as the prescription of nutritional supplements via oral or enteral nutrition. These approaches facilitate the necessary initial increase in food volume to promote weight gain, without immediately incorporating new or avoided foods into the diet [26, 71, 92, 93].

AN and ARFID share common challenges such as malnutrition, nutrient deficiencies, and psychosocial impairment resulting from restrictive eating behaviours. While these disorders differ in their precipitating and sustaining factors, the treatment of both conditions involves similar components, including the normalization of weight and eating behaviour as therapeutic targets [26, 91]. However, patients with ARFID and AN may have distinct therapeutic needs regarding dietary and nutritional treatment, and the dietary prescription may vary in terms of quality, quantity, and food choices. For instance, it is known that patients with ARFID tend to reject foods that are considered "safe" by individuals with AN while preferring those deemed "dangerous" by AN patients. This can present a challenge when implementing the same treatment strategies and environmental structures for both disorders [9].

One of the main objectives is to adapt the eating routine or supplementary to provide all macro and micronutrient needs. When avoiding and restricting specific consumption of cereals and grains, it will be necessary to pay attention to the recovery of Carbohydrates and Fiber; when restricting animal products, legumes and dairy products, pay attention to the intake of Protein (relevant in the process of malnutrition) and Riboflavin/Vitamin B2, Cobalamin/vitamin B12, Iron, Selenium and Zinc; when restricting fish and seafood, pay attention to the recovery of Vitamin D and Omega-3 acids; in fruits and vegetables, pay attention to Vitamin C, folates and other minerals; and finally, when restricting animal and fruit/vegetable fats, pay attention to recovering the intake of Fats and Vitamin E, Vitamin A, Vitamin K [94].

In a hospital or outpatient setting, the nutritionist or dietitian, as an integral part of the multidisciplinary team, typically calculates the dietary therapy prescription, estimates energy and fluid requirements for oral intake, assesses the adequacy of macro and micronutrients, and plans meal schedules. Furthermore, the nutritionist is responsible for establishing goals for nutritional therapy, defining a plan for monitoring the dietary therapy prescription, which includes assessing nutritional and hydration status, tracking progress in oral intake, adjusting oral and enteral feeding when necessary, and providing appropriate guidance on nutritional needs while developing an individualized meal plan [93, 95–98].

Another objective is to reintroduce of new foods or those previously avoided and this is gradually integrated into the individual's routine through exposure experiences led by professionals from the multidisciplinary team. Notably, the severity of food refusal observed in individuals diagnosed with ARFID increases the likelihood of requiring enteral nutritional support, in comparison to those with AN [6, 84, 92]. Within specialized treatment centres, the use of enteral nutrition is a common practice for cases of restrictive eating disorders. However, during the initiation of the refeeding protocol, close monitoring for electrolyte abnormalities, which may arise from refeeding syndrome—a serious and potentially fatal complication—is essential [26, 95]. Protocols and guidelines have been proposed and are widely utilized in clinical practice to safely commence refeeding in severely malnourished individuals with eating disorders or those who have experienced prolonged periods of starvation [93, 96, 99–104].

Considering that long-term dependence on enteral nutritional support may be a component of the diagnostic criteria for ARFID, enteral administration is regarded as a temporary measure as part of the treatment. However, caution is required when opting for its use, as it can lead to iatrogenic effects such as reduced expectations regarding the consumption of solid foods, hindering the development of sensory skills related to eating, gastrointestinal symptoms, and other discomforts associated with food intake [6, 90, 105]. Once the patient's nutritional and fluid needs are met, leading to clinical stabilization, other therapeutic strategies can be initiated. These may include nutritional counselling, specific psychological interventions, restoration of healthy dietary patterns that facilitate social interactions and well-being, desensitization to avoided foods, and the introduction of new foods into the diet [30, 97, 105, 106].

And finally, nutritional counselling plays a vital role in addressing dysfunctional eating behaviours in eating disorders. Its primary objective is to guide individuals towards adopting a normal diet across all dimensions, thereby restoring physical and psychological health. In children and adolescents, this process also aims to facilitate age-appropriate development. To achieve this, nutritionists provide guidance, support, and strategies to prepare individuals for the challenges they may encounter during treatment, such as behavioural and emotional difficulties, gastrointestinal discomfort, changes in body composition, and potential medical issues [93]. Signs and symptoms of specific vitamin-mineral deficiencies could be invetigated due to group of dietary restrictions and supplemented or reinserted in different preparations [94]. Another fundamental tool in the treatment of eating disorders is the development of a food plan by a nutritionist. This plan offers practical, organized, and tailored guidance on energy and nutrient requirements, with gradual modifications. Initially, the focus is on meeting energy needs, followed by an emphasis on macronutrients and micronutrients [93].

### Pharmacological intervention in ARFID

Currently, there is limited empirical evidence regarding pharmacological treatments for the management of ARFID. However, case reports and small case series studies have explored the use of certain psychotropic drugs as adjunctive therapeutic interventions for ARFID and its associated psychiatric comorbidities [26, 95].

Olanzapine, a second-generation atypical antipsychotic, has been associated with reduced cognitive rigidity in beliefs about food and increased appetite and subsequent weight gain [26, 89]. Its mechanism of action involves blocking histaminergic and serotonergic receptors in the lateral hypothalamus, thereby stimulating food intake [107–109]. Clinical research examining the effects of olanzapine on weight recovery in patients with AN has yielded modest results, demonstrating an association between olanzapine use and gradual weight gain in AN patients over time [110–113]. A case study analyzed the use of low-dose olanzapine as an adjunct to conventional treatment, including individual, group and family therapy, nutritional counselling, and pharmacotherapy, in an eating disorder program for children and adolescents. The study compared individuals who received olanzapine with those who did not and found a statistically significant difference in weight gain. Adjuvant olanzapine also helped reduce associated anxiety, depressive symptoms, and cognitive impairments [26, 95].

Another psychotropic drug of interest is Mirtazapine, an antidepressant known for its safety and efficacy in treating depressive and anxious symptoms in adults. The rationale for its use in ARFID is its antagonistic activity on H1 histaminergic receptors, which can promote an orexigenic effect, induce weight gain, and increase gastric emptying [114–117]. Gray et al. (2018) reported the use of mirtazapine to enhance appetite and facilitate weight gain while reducing nausea and vomiting [118]. The study observed an average weekly change in BMI of 0.10 before starting mirtazapine and an average weekly change in BMI of 0.23 after mirtazapine treatment, suggesting that mirtazapine may promote weight gain in patients with ARFID. Mirtazapine was found to be well tolerated and associated with a higher rate of weight gain compared to standard weight restoration programs [15, 118]. Tanidir and Herguner (2015) described the use of mirtazapine in the treatment of a pediatric patient with food avoidance due to an aversive eating experience and diagnosed with ARFID. The patient exhibited a reduction in anxiety and fear symptoms, increased appetite, and weight gain following the initiation of mirtazapine treatment [119].

Buspirone, which has shown efficacy in treating Generalized Anxiety Disorder, has also been explored as a potential treatment for anxiety symptoms in adolescents with ARFID. A case report described the successful use of buspirone in reducing anxiety symptoms, improving eating behaviours by reducing fear of vomiting, and promoting weight gain in a female adolescent with ARFID resulting from an aversive eating experience [15, 120]. This case study suggests further investigation into the potential usefulness of buspirone in ARFID treatment [120, 121]. Additionally, a case series detailed six patients diagnosed with ARFID who received treatment in a hospital-based eating disorder program. The treatment approach included family therapy, cognitive-behavioural therapy, medical monitoring, and pharmacological treatment with olanzapine, fluoxetine, and cyproheptadine (used as an appetite stimulant in two cases). After treatment, all six patients achieved the desired BMI [122].

There is currently no psychotropic medication for treatment of ARFID approved by the U.S. Food and Drug Administration. Future placebo-controlled randomized clinical trials are necessary to establish the efficacy of these pharmacological interventions as potential strategies for the treatment of ARFID [26].

### Behavioral intervention in ARFID

Psychotherapeutic approaches to managing ARFID, along with the aforementioned therapeutic modalities, necessitate an individualized assessment of restrictive behaviours. This assessment serves as the basis for developing a tailored plan to address specific avoidant and restrictive behaviours and expand the consumption of diverse food groups [5].

While treatment protocols similar to those used for other eating disorders, such as AN, have been employed for ARFID, it is important to recognize that ARFID represents a distinct clinical entity. Therefore, adopting identical therapeutic protocols is not considered sufficient or recommended, as ARFID patients exhibit unique psychopathology and present distinct predisposing, precipitating, and maintaining factors (in addition to not always being underweight) [66].

Solid evidence on effective treatments for ARFID is limited. Nonetheless, findings from single case studies, case series, and non-randomized clinical trials employing psychological interventions show promising results in reducing ARFID symptoms. Cognitive-Behavioural Therapy (CBT) adapted for ARFID has been increasingly studied as a potential intervention [123–128], as well as the utilization of Family-Based Therapy (FBT) as a treatment strategy [122, 125, 129].

A case report by King, Urbach, and Stewart (2015) highlighted the successful treatment of a patient with limited food intake due to fear of aversive consequences using a cognitive-behavioural therapy approach. Positive outcomes included weight gain, increased daily caloric consumption, improvements in cognition and energy levels, and a general reduction in anxiety [124].

Dumont et al. (2019) presented a clinical case series involving eleven patients aged between 10 and 18 years who received four weeks of daily cognitive-behavioural therapy specifically designed to target the causal factors of food restriction and avoidance in adolescents with ARFID. This protocol incorporated inhibitory learning principles and repeated exposures to food stimuli, demonstrating preliminary success in treating various clinical presentations of ARFID by reducing symptoms, such as increased food acceptance, reduced anxiety, and decreased psychosocial interference. Tube feeding was discontinued in six out of the eleven patients observed [123].

Thomas et al. (2020) evaluated the feasibility, acceptability, and preliminary efficacy of Cognitive-Behavioral Treatment for ARFID (CBT-AR) in individuals aged 10 years and older, encompassing all clinical presentations of ARFID. Preliminary results indicated a significant reduction in symptoms and ARFID severity scores on the PARDI. Patients also exhibited increased variety in their food consumption, incorporating new foods into their routines following treatment, and weight gain was observed in underweight patients [127].

In an adult clinical sample, Thomas et al. (2021) conducted a proof-of-concept study on the use of CBT-AR, which yielded promising results and justified further investigation into the safety and efficacy of this approach [128]. Significant decreases in ARFID severity scores on the PARDI and sensory sensitivity were observed, along with notable weight gain. However, anxiety and depression levels did not show significant changes.

Family-Based Therapy (FBT), commonly utilized in the treatment of eating disorders in adolescents, particularly for AN and BN, focuses on educating and training caregivers to support patient recovery in an outpatient setting [125, 130]. FBT places emphasis on modifying eating behaviours through caregiver management, recognizing them as key agents in driving change in dysfunctional eating behaviours [122, 125].

Lock et al. (2018) presented three case reports illustrating the application of family-centred treatment adapted for ARFID (FBT-ARFID) in preadolescents diagnosed with the disorder. These reports demonstrated significant clinical progress in reducing symptoms, including weight gain, increased food variety in the diet, and decreased anxiety symptoms. The authors suggested that such an intervention could be employed for ARFID, considering the unique clinical presentations of patients. While the available data is limited and should be interpreted with caution due to methodological limitations such as study design, small sample sizes, and short follow-up periods, the findings suggest that FBT-ARFID may be a viable, potentially promising, and effective approach for this population [122, 125, 130].

## Future directions for research

Further epidemiological studies are required to investigate the trajectory of ARFID over time, contributing to a more comprehensive understanding of the mechanisms underlying food restriction and avoidance, as well as the etiology, risk factors, and therapeutic interventions. Patients with ARFID exhibit distinct biological foundations, thus a thorough characterization of the various clinical presentations is crucial for developing a better conceptual understanding. This understanding will aid in determining the implications of these phenotypes in terms of diagnosis, treatment, course, and prognosis, which may vary for each patient. Additionally, it can provide guidance for the necessary and appropriate care of such individuals.

Prospective and long-term follow-up studies are necessary to comprehend the course of the disorder and the potential impairments in the development of physical and social skills. Limited knowledge of avoidant and restrictive eating hampers effective treatment and management, with long-term consequences for the growth, development, nutrition, and psychosocial functioning of children and adolescents.

Moving forward, several areas of research are particularly intriguing. One such area is the validation of screening instruments that are sensitive to the heterogeneity of clinical characteristics. While initial evidence supports the sensitivity and validity of existing screening and diagnostic tools, larger-scale studies are required to test their psychometric properties in more extensive samples and cohorts from diverse clinical settings over extended observation periods. Future investigations could also delve into the neurobiology associated with ARFID, thereby contributing to a greater understanding of the processes and mechanisms underlying food avoidance and its perpetuation in children, adolescents, and adults. Although significant progress has been made in understanding the pathogenesis of ARFID, further studies are needed to examine the long-term outcomes and consequences of delayed diagnosis.

Furthermore, in-depth research into existing neurobiological models for ARFID is crucial. This disorder involves alterations in sensory perception, sensory hypersensitivity, dysregulation of appetite homeostasis, and differences in the activation of regulatory centres for hunger and satiety perception. Additionally, excessive activity in negative valence systems that correlate with phobic characteristics has been observed. Exploring the neurobiological bases and hypotheses that have already been proposed may yield promising avenues for comprehending the etiology, pathophysiology, and personalized treatment of ARFID.

Moreover, it is important to evaluate the outcomes and efficacy of the various therapeutic interventions that have been proposed for the specific characteristics of ARFID. Rigorous randomized clinical trials, incorporating reliable control conditions and standardized measures, are needed to determine which treatments or combinations of treatments are effective for different presentations and phenotypes of ARFID, as well as their responsiveness to various therapeutic option. Lastly, educational and awareness programs targeting health professionals are of utmost importance to promote best practices in the care and treatment of this relatively unknown and highly challenging eating disorder.

## Conclusions

ARFID is a relatively recent diagnostic classification, representing a burgeoning field of study. The identification of diagnostic criteria and the pursuit of new knowledge in this area have only recently begun. Consequently, assessment tools and treatment strategies are still in the process of development and validation. This narrative review explored the neurobiological perspective of ARFID using the three-dimensional model, examined its etiology and risk factors, evaluated clinical screening and evaluation tools, discussed common clinical complications, and presented different types of nutritional, behavioural, and pharmacological interventions used in ARFID treatment.

## Data Availability

Not applicable.
